# Low-dose olanzapine for cancer-associated anorexia and nausea: insights from clinical practice

**DOI:** 10.3332/ecancer.2026.2054

**Published:** 2026-01-07

**Authors:** Rituparna Biswas, Krishnangshu Bhanja Choudhury, Anirban Halder

**Affiliations:** 1 Department of Radiation Oncology, Malda Medical College, Malda 732101, India; 2 Department of Radiation Oncology, Manipal Hospital Dhakuria, Kolkata 700029, India; a https://orcid.org/0000-0002-2585-3283; b https://orcid.org/0000-0002-4336-1485; c https://orcid.org/0000-0002-2721-5031

**Keywords:** cancer cachexia, anorexia, olanzapine, nausea, supportive care

## Abstract

**Background:**

Cancer anorexia-cachexia syndrome (CACS) is a multifactorial metabolic condition prevalent among patients with advanced malignancies and often exacerbated by chemotherapy or radiotherapy (RT). While pharmacologic options such as megestrol and corticosteroids are available, their use is limited by cost or adverse effects. Olanzapine, a second-generation antipsychotic, has recently been recommended by American Society of Clinical Oncology for managing CACS, but real-world data remain scarce.

**Methods:**

This retrospective cohort study was conducted at a tertiary oncology centre in West Bengal, India, and included patients aged 18–70 years with any solid malignancy and severe anorexia, receiving chemotherapy, RT or palliative care. All patients were treated with low-dose Olanzapine (2.5 mg/day) for 12 weeks. Data were extracted from medical records for the period between 1 January 2024, and 31 January 2025.

**Results:**

Fifty patients met the inclusion criteria. The median age was 44.5 years and 82% had Stage III/IV disease. Of these, 82% (*n* = 41/50) reported improvement in anorexia symptoms, 82% maintained or gained weight and 16% (*n* = 8/50) gained at least 1 kg. Among 24 patients with refractory nausea, 50% reported symptomatic relief. No adverse events attributable to Olanzapine were documented.

**Conclusion:**

Low-dose olanzapine (2.5 mg/day) is an effective, well-tolerated and cost-efficient option for the management of cancer-related anorexia and nausea in real-world clinical settings. Its use may be particularly beneficial in resource-limited environments and should be considered as a first-line pharmacologic intervention for CACS. Further prospective studies are warranted.

## Introduction

Cancer anorexia- cachexia syndrome (CACS) is a multifactorial metabolic condition characterised by reduced appetite, unintended weight loss, muscle wasting and functional decline [1, 2]. It affects up to 70%–80% of patients with advanced malignancies, particularly those with gastrointestinal, pancreatic and lung cancers [3]. Chemotherapy and radiotherapy (RT) may further induce mucositis, dysphagia or nausea, which may aggravate anorexia. This syndrome significantly impairs quality of life, reduces tolerance to anticancer therapies, and is associated with poor clinical outcomes, including shortened survival [4].

Despite the high prevalence and clinical burden of CACS, effective therapeutic options remain limited. Current clinical guidelines recommend dietary counselling as a first-line approach to address cancer-associated anorexia and weight loss. However, the effectiveness of pharmacologic agents remains limited and often unsatisfactory. Megestrol acetate, a synthetic progestin, has demonstrated modest improvements in appetite and weight gain but is associated with significant risks, including thromboembolic events and even increased mortality in frail populations. Glucocorticoids can provide temporary appetite stimulation and enhanced well-being; however, their benefits typically are transient and long-term use is limited by adverse effects such as hyperglycaemia, immunosuppression and muscle wasting [5].

Olanzapine, an atypical antipsychotic agent with antagonistic effects on multiple neurotransmitter receptors—including dopamine (D1–D4), serotonin (5-HT2A/2C/3), histamine (H1) and muscarinic receptors—has emerged as a promising candidate in the management of CACS [6]. Originally observed to cause significant weight gain in psychiatric populations, olanzapine has demonstrated appetite-stimulating effects that may be therapeutically beneficial in oncology settings. It is already widely utilised as an antiemetic in chemotherapy-induced nausea and vomiting and has shown potential in alleviating refractory nausea and anorexia in palliative care populations [7–10].

Following the publication of results by Sandhya *et al* [6], the American Society of Clinical Oncology (ASCO) updated its clinical guidelines to recommend the use of low-dose continuous olanzapine for improving appetite and promoting weight gain in patients with advanced cancer. However, its real-world effectiveness is yet to be established.

This study aims to evaluate the efficacy of low-dose olanzapine in improving anorexia, mitigating weight loss and tackling refractory nausea among patients receiving active oncologic therapy and those under best supportive care. By focusing on a pragmatic, low-cost intervention with a favourable safety profile, we hope to contribute to the evolving management strategies for cancer-associated anorexia and cachexia.

## Methods

### Study design and setting

A retrospective cohort study was conducted at the Oncology department of our institution (a Medical College in West Bengal, India).

### Inclusion criteria

Patients with any solid malignancy undergoing chemotherapy or RT or only palliative care (no active anti-cancer treatment), having severe anorexia, were included in the study. Severe anorexia was defined as marked subjective loss of appetite, prohibiting adequate food intake and causing discomfort to the patient. Age limit of 18–70 years was permitted. Patients who were on alternate Psychotropics or on long-term steroids were excluded as it might have an additional orexigenic effect. However, a short course of steroids or Olanzapine (5–10 mg) as part of an anti-emetic policy was allowed.

### Treatment

Patients with significant anorexia who cannot afford Megestrol acetate or having contraindications are routinely administered low-dose Olanzapine (2.5 mg) per oral daily at bedtime for 12 weeks. Moreover, Olanzapine is dispensed free of charge through the hospital pharmacy, ensuring uniform access to the same brand and eliminating additional financial burden.

### Assessment & data collection

Patients receiving chemotherapy are routinely assessed for treatment-related toxicities prior to each subsequent cycle, whereas those undergoing RT are evaluated on a weekly basis in our department. Following the completion of treatment, patients are monitored according to predefined follow-up schedules. Individuals receiving palliative care alone are called at regular weekly or monthly intervals. These review data are meticulously recorded routinely in our department. Data for this study were retrospectively collected through a review of patients' medical records. Only those with complete follow-up data for a minimum of 12 weeks, between 1 January 2024, and 31 January 2025, were included in the analysis.

## Results

### Baseline characteristics

A total of 50 patients who met the eligibility criteria during the study period were retrospectively reviewed. Baseline demographic and clinical characteristics are summarised in Table 1. The median age of the cohort was 44.5 years, with males comprising 54% of the study population. A significant proportion of patients (82%) had advanced-stage disease (Stage III or IV). Nearly all anatomical cancer subsites were represented. In terms of treatment, most patients were receiving chemotherapy or RT, while 24% were managed with best supportive care alone, without active oncologic intervention.

### Observations

**Figure 1.**
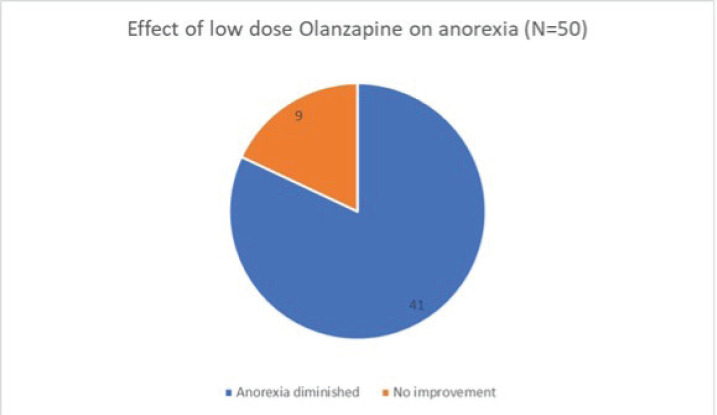
Effect of low-dose Olanzapine on anorexia.

**Figure 2.**
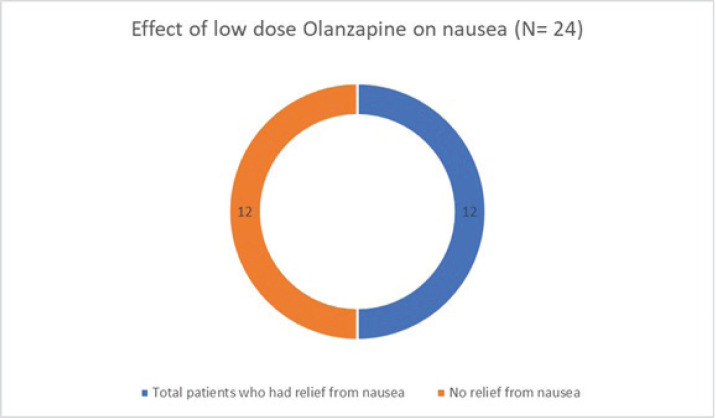
Effect of low-dose Olanzapine on nausea.

**Figure 3.**
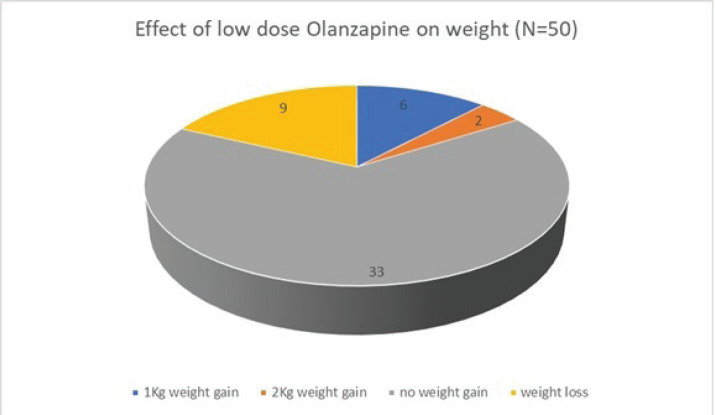
Effect of low-dose Olanzapine on weight.

Of the 50 patients with severe anorexia at baseline who completed a twelve-week course of low-dose Olanzapine, a whopping 82% (41 out of 50) reported diminution of symptoms (Figure 1). Amongst the study population, 24 patients had intractable nausea despite standard antiemetic measures; of these, 50% reported relief following treatment with low-dose olanzapine (Figure 2). Given the retrospective nature of the study, formal grading of symptom resolution was not feasible; instead, a subjective improvement in anorexia and nausea, as documented in medical records, was considered a favourable response. Notably, 82% of patients sustained no further weight loss; in fact 16% experienced a weight gain of at least 1 kg as evident in Figure 3. No untoward adverse events pertaining to Olanzapine use were documented in the medical records.

## Discussion

CACS remains a critical challenge in oncologic care due to its multifactorial nature, high prevalence and deleterious impact on patient outcomes. This retrospective study assessed the real-world effectiveness of low-dose Olanzapine (2.5 mg/day) in improving anorexia, preventing further weight loss and controlling refractory nausea in patients receiving cancer treatment or best supportive care.

Our findings align with those of **Sandhya *et al*** [6], who demonstrated in a randomised, double-blind, placebo-controlled trial that low-dose olanzapine significantly improved appetite (43% versus 13%), promoted weight gain (>5% in 60% versus 9%) and enhanced quality of life compared to placebo among patients with upper GI and lung cancers undergoing chemotherapy. In our cohort, 82% of patients experienced symptomatic relief from anorexia and 16% achieved weight gain, despite receiving Olanzapine in diverse clinical settings and cancer types. Notably, our population also included patients under palliative care, extending the scope of evidence for Olanzapine use.

The current study also complements data from **Okamoto *et al*** [11], who observed a 149% relative increase in food intake among patients with cancer-related anorexia following low-dose olanzapine, even in those without concurrent nausea or vomiting. They reported Olanzapine's ameliorating efficacy in cancer-related anorexia at further low doses of 1.5 mg/day [11].

In contrast, the **phase I trial by Naing *et al*** [2] reported only a modest and statistically non-significant trend toward weight stabilisation with Olanzapine in patients with advanced cancers having a weight loss ≥ 10% of body weight over 6 months. Furthermore, metabolic cytokine changes (e.g., IL-6, leptin and ghrelin) did not correlate with weight gain in that study, highlighting the complexity of CACS pathophysiology.

Our study found a 50% response rate in patients with refractory nausea—like antiemetic efficacy observed in an earlier study by **Kaneishi *et al*** [10], supplemented by other trials and systematic reviews, such as those by **Chow *et al*** [7] and **Chelkeba *et al*** [8], which established Olanzapine’s broad antiemetic utility. This dual benefit—amelioration of both anorexia and nausea—makes Olanzapine particularly attractive in oncology, where symptom overlap is common.

Importantly, **Navari and Brenner** [3] showed that combining Olanzapine with Megestrol acetate led to significantly higher appetite improvement and weight gain than megestrol alone. While megestrol remains an established agent, its risk profile, particularly thromboembolic events, often limits use in frail populations. Olanzapine, conversely, was well tolerated in our study, with no reported adverse events. The tolerability and low cost—especially when dispensed freely in resource-limited settings—enhance its feasibility for wider implementation.

A 2024 review by **Poon *et al*** [4] supported these findings and emphasised the recent **ASCO recommendation** endorsing low-dose Olanzapine for cancer cachexia, citing a favourable safety and efficacy profile. The authors underscored the need for clinicians to tailor use based on patient performance status, nutritional goals and care setting—considerations mirrored in our pragmatic, retrospective design.

Despite these encouraging results, limitations exist. The retrospective nature and absence of a control arm limit causal inference. Appetite improvement was assessed subjectively and nutritional status was not quantified using validated scales. Moreover, biochemical markers (e.g., cytokines, leptin and ghrelin) were not evaluated. Nonetheless, this study adds valuable real-world evidence supporting low-dose Olanzapine as a safe, accessible and effective intervention for cancer-associated anorexia.

## Conclusion

This retrospective study reinforces the role of low-dose Olanzapine (2.5 mg/day) as a safe, affordable and effective intervention for managing cancer-related anorexia and associated symptoms. A significant proportion of patients experienced symptomatic relief, stabilisation of weight and improvement in refractory nausea without notable adverse effects. These findings complement and extend prior controlled trials by demonstrating real-world applicability across diverse cancer types and treatment settings, including palliative care.

Given its favourable safety profile, ease of administration and cost-effectiveness, low-dose olanzapine should be prioritised, particularly in settings where access to other agents such as megestrol is limited or contraindicated.

## Conflicts of interest

The author(s) declare that they have no conflicts of interest.

## Funding

Not applicable.

## Consent for publication

Written informed consent has been taken from patients/relatives.

## Author contributions

RB and KBC contributed to the conception of the study, data collection & evaluation. SKC and AH prepared the manuscript. RB, KBC, SKC & AH substantively revised it. All authors have read and approved the manuscript.

## Figures and Tables

**Table 1.** Baseline characteristics.

Characteristics	*N* = 50
**Median age in years**	44.5 (Range = 26–65 years)
**Sex**	
Male	27 (54%)
Female	23 (46%)
**Stage of cancer (AJCC 8th edition)**	
1	3 (6%)
2	6 (12%)
3	18 (36%)
4	23 (46%)
**Types of cancer**	
GI + HPB	14 (28%)
Breast	8 (16%)
Head & neck + skin	11 (22%)
GU	5 (10%)
Gyn	6 (12%)
Others	6 (12%)
**Management**	
Chemotherapy or radiotherapy	38 (76%)
Palliative care only	12 (24%)

*Abbreviations:* AJCC - American Joint Committee on Cancer, GI - Gastrointestinal cancer, HPB- Hepatopancreaticobiliary cancer, GU - Genitourinary, Gyn - Gynaecological cancer
